# Esophageal cancer occurrence in Southeastern Iran

**Published:** 2010

**Authors:** Bijan Ziaian, Vahid Montazeri, Reza Khazaiee, Shahram Amini, Mehrbod Karimi, Davood Mehrabani

**Affiliations:** a Department of General Surgery, Shiraz University of Medical Sciences, Shiaraz, Iran; b Department of Pathology, Zahedan University of Medical Sciences, Zahedan, Iran; c Gastroenterohepatology Research Center, Department of Pathology, Shiraz University of Medical Sciences, Shiraz, Iran

Esophageal cancer is globally considered as the sixth most common cancer with annual new cases of about 412,000. The mortality rate due to esophageal cancer was globally reported as 337,500 out of all 6.2 million cancer deaths in 2000.^1^ The highest incidences were reported in China, the Caspian region of Iran, South Africa, and France.^2^ In southern Iran, esophageal cancer ASR is 1.05 in males and 0.87 in females with more incidence in subjects older than 60 years old.^3^ This active hospital-based study aims to determine the characteristic of esophageal cancer in southeastern Iran.

The Zahedan Hospital-based Cancer Registry of Department of Surgery is the only registry center in South-eastern Iran based on its equipped Khattam-al-Anbia General Hospital which is affiliated to Zahedan University of Medical Sciences and it’s due to the presence of cancer specialists in the department. Between 1995 and 2007, data were provided based upon ICD-0 and registered cases included all invasive cancers in ICD-10 categories of C-00 to C-80 and all duplicate cases were removed. Our registry personnel interviewed all patients to record the information face-to-face.

Nine hundred and two patients were registered as cases of esophageal cancer including 43.2% males and 66.8% females with a male to female ratio of 1/1.3. Two cases were less than 30 years old with a previous history of caustic esophageal injury. Most patients were between 60 to 80 years old (60%) while 4 patients were older than 80 years; 75.6% (682 cases) of esophageal cancers were squamous cell carcinoma (SCC) and 24.4% (220 cases) were adeno carcinoma. Of all, 1.4% of SCCs were observed in upper third part of esophagus, 75.6% in the middle third and 23% in the lower third. For adenocarcinoma, the figures were 0.0%, 9.1% and 90.9% respectively. All patients suffered from dysphagia. Weight loss was visible in 91.4% of cases and anorexia in 26.3%. In relation to predisposing factors, 94% reported a previous history of drinking hot tea, naswar (which is tobacco mixed with ash and kept in the buccal cavity between the lips and the alveolus) consumption in 83% of cases, opium abuse in 70% and smoking in 36% of subjects. Surgery was the main method of treatment performed together with radiotherapy (in cervical cancers and in SCCs) and chemotherapy with 5FU and cisplatin.

Esophageal cancer was more in women of the present study which is different from other studies in the US and Pakistan which may be due to more exposure to the risk factors.^4^,^5^ Badar et al reported an increased risk of SCC in the lower-third of the organ^6^ which is similar to the results of the present study. In Pakistan, China and western countries, it was shown that smoking, eating naswar and inhaling substances were high risk factors among esophageal cancer patients.^7^–^9^ Eating and drinking hot food and beverages was previously reported as the risk factors for esophageal cancer which can explain the present results too.^10^ The present results demonstrated a higher prevalence of esophageal cancer in females, age group of older than 60 years and smokers and a most common type of squamous cell carcinoma. Therefore, these results may help the authorities in their health planning programs. More studies in a longer time period are necessary to explain the causes.
